# Withdrawal Time Estimation and Dietary Risk Assessment of Sulfamethoxazole in GIFT Tilapia (GIFT *Oreochromis niloticus*) After Oral Administration

**DOI:** 10.3390/vetsci12060598

**Published:** 2025-06-18

**Authors:** Xinyue Wang, Ruiqi Fan, Saisai Wang, Yuanyuan Ren, Xin Zhang, Yingchun Mu, Sudong Xia, Xiaoyu Wang, Bo Cheng

**Affiliations:** 1 College of Fisheries, Tianjin Agricultural University, Tianjin 300392, China; 2 Key Laboratory of Quality and Safety Control of Aquatic Products, Ministry of Agriculture and Rural Affairs, Chinese Academy of Fishery Sciences, Beijing 100141, China; 3 Fisheries Science Institute, Beijing Academy of Agriculture and Forestry Sciences, Beijing 100068, China

**Keywords:** sulfamethoxazole, residue, elimination, withdrawal period, antibiotics, health risk

## Abstract

Sulfamethoxazole (SMZ), a widely used broad-spectrum antibiotic in aquaculture, lacks comprehensive research on its residual elimination kinetics in tilapia. This study investigated SMZ residue depletion, withdrawal periods, and dietary risks in 1-year-old GIFT tilapia (Genetically Improved Farmed Tilapia *Oreochromis niloticus*) weighing 500 ± 50 g, following oral gavage administration of a loading dose (200 mg/kg BW on day 1) and then 100 mg/kg BW daily for 6 more days, at 22 ± 2 °C. Tissue samples (plasma, muscle, skin, liver, kidney, gill, and remaining tissues) were collected from five fish per time point at intervals from 0.33 to 30 days post-administration, with SMZ residues quantified via HPLC-MS/MS. Results revealed peak SMZ concentrations at 0.33 days (8 h), ordered as liver > skin > plasma > kidney > remaining tissues > gill > muscle. Muscle residues fell below the maximum residue limit (MRL, 100 μg/kg) by day 3, while skin required 10 days. Kidney residues dropped below the limit of detection (LOD) earliest (16 days), followed by muscle, gill, and remaining tissues (25 days), whereas plasma, liver, and skin retained detectable levels until day 30. Elimination equations for SMZ across tissues exhibited first-order kinetics. Based on the specific conditions of this study, a minimum 11-day withdrawal period is recommended for edible tissues (muscle + skin) after SMZ administration. Hazard quotient (HQ) values for all tissues remained below the safety threshold (HQ = 1), indicating low dietary risk. These findings support SMZ use standardization in tilapia aquaculture to ensure food safety compliance.

## Simple Summary

Sulfamethoxazole is a commonly used antibiotic in fish farming, but residues from this antibiotic in fish can pose health risks to consumers if not managed correctly. This study investigated how long it takes for sulfamethoxazole residues to eliminate from tilapia, a popular farmed fish species, and aimed to establish safe guidelines for both aquaculture producers and consumers. Researchers provided tilapia with a controlled antibiotic diet for seven days and then monitored residue levels in various parts of the fish, such as muscle, skin, and organs, over a period of 30 days. Results indicated that antibiotic residues decreased to safe levels within three days in muscle tissue, while skin required ten days to reach safety standards. However, some tissues retained traces of the antibiotic up to 30 days. Consequently, to ensure consumer safety, aquaculture producers are advised to wait at least 11 days after antibiotic administration before harvesting the fish for market. The findings confirm that fish treated according to these guidelines are safe for human consumption. This research helps aquaculture producers responsibly manage antibiotic use, protects public health, and promotes sustainable aquaculture, ultimately ensuring safe and healthy seafood for consumers.

## 1. Introduction

The culture of Nile tilapia (*Oreochromis niloticus*) can be traced to ancient Egyptian times as depicted on bas-relief from an Egyptian tomb dating back over 4000 years. Due to its advantageous traits, including rapid growth, omnivorous feeding habits, robust disease resistance, and lack of intermuscular spines, the Food and Agriculture Organization of the United Nations (FAO) designated Nile tilapia as a globally recommended aquaculture species in 1976 [1]. Further enhancing the production potential of Nile tilapia, the Genetically Improved Farmed Tilapia (GIFT) project (1988–1997), a collaboration using selective breeding on wild Nile tilapia, developed superior strains exhibiting an 85% increase in growth rate [2]. Tilapia ranks second in global freshwater fish production, and the global total production reached 5.3 million tons in 2022 [3]. Currently, China has the largest scale of tilapia farming globally, according to the “China Fishery Statistical Yearbook 2024” [4]. However, with the continuous expansion of farming scale and increased stocking density, bacterial diseases caused by *Streptococcus agalactiae*, *Pseudomonas fluorescens*, *Edwardsiella* spp., and other pathogens have become increasingly prevalent [5,6], posing a significant challenge to the sustainable development of the tilapia aquaculture industry [7].

Sulfonamides are synthetic antibacterial agents that inhibit bacterial folic acid metabolism, and that have widespread application in animal husbandry and aquaculture [8,9]. Among these, sulfamethoxazole (SMZ) is frequently employed in both marine and freshwater settings due to its broad-spectrum activity [10,11,12]. However, the residues of antibiotics in animal-derived foods may pose potential risks to human health, including allergic reactions, teratogenic effects, and carcinogenicity [13]. Consequently, regulatory bodies worldwide, including those in the United States, China, and Europe, strictly monitor sulfonamide residues, and have established a harmonized maximum residue limit (MRL) of 100 μg/kg in fish tissue [14,15,16].

Currently, four sulfonamide preparations are approved for aquaculture use in China, including those containing sulfamonomethoxine, sulfadiazine, sulfamethoxazole, and sulfamethazine [17]. The “Code of Practice for the Use of Veterinary Drugs in Fisheries” (SC/T 1132-2016) recommends a dosage of compound sulfamethoxazole for freshwater-cultured fish of 100 mg/kg [18], administered twice daily for 5–7 consecutive days, with an initial doubled dose, and a uniform withdrawal period of 500 degree-days for all fish. However, recent studies have revealed significant species-specific differences in the metabolism of sulfonamides in aquatic animals. Even within the same species, factors such as age and gender may influence drug metabolism [19,20,21]. Given the global diversity of aquaculture species, it is essential to establish species-specific guidelines for veterinary drug use. Moreover, the widespread occurrence of indiscriminate drug use and overdosage in the industry often leads to excessive sulfonamide residues in tilapia and other aquatic products, posing serious risks to food safety and hindering the international trade of aquatic products [22,23].

To address these issues, this study focuses on tilapia and SMZ, simulating high-dose and high-frequency usage patterns commonly encountered in production settings. The study aims to systematically investigate the elimination and metabolism of SMZ residues in six different tissues of tilapia. However, given the limited information on SMZ metabolites in tilapia, the analysis in this study was currently limited to the parent compound, SMZ. The findings will provide a scientific basis for optimizing the use of SMZ and establishing appropriate withdrawal periods for aquaculture production. This study is therefore significant as it provides the first comprehensive dataset on SMZ depletion in commercially critical GIFT tilapia under a simulated high-dose regimen. Such species-specific data are essential for replacing one-size-fits-all regulations with evidence-based withdrawal periods that safeguard public health.

## 2. Materials and Methods

### 2.1. Pharmaceuticals and Reagents

The SMZ standard (≥99.0%) and sulfadoxine-D3 (99.8% ± 0.2%) were both purchased from Dr. Ehrenstorfer GmbH (Augsburg, Germany). The raw material of SMZ (≥99.0%) was purchased from Elan Chemical Technology Co., Ltd. (Shanghai, China). Disodium hydrogen phosphate (analytical grade), sodium dihydrogen phosphate (analytical grade), sodium chloride (analytical grade), perchloric acid (analytical grade), and sodium bicarbonate (analytical grade) were all purchased from Sinopharm Chemical Reagent Co., Ltd. (Shanghai, China). Methanol (chromatographic grade) and acetonitrile (chromatographic grade) were purchased from Thermo Fisher Scientific, Inc. (Waltham, MA, USA) Formic acid was purchased from CNW Technologies GmbH (Dusseldorf, Germany), and ammonium acetate was purchased from Fluka (Morris Plains, NJ, USA).

### 2.2. Instruments and Equipment

The instruments and equipment used in the experiment were as follows: a TSQ Quantum liquid chromatography-mass spectrometry (LC-MS) instrument (Thermo Fisher Scientific, Inc., Waltham, MA, USA); an HCB 1002 electronic balance (Adam Equipment Co., Ltd., Wuhan, China); an H-2050R centrifuge and H/T16MM centrifuge (Hunan Xiangyi Laboratory Equipment Development Co., Ltd., Changsha, China); an N-EVAP1/2 nitrogen evaporator (Organomation Associates, Inc., Berlin, MA, USA); an MS3 vortex mixer (IKA Works, Inc., Wilmington, NC, USA); an SB-5200DTN ultrasonic cleaner (Xinzhi Co., Ltd., Ningbo, China); and a Milli-Q ^®^ desktop pure water system (Millipore Sigma, Billerica, MA, USA).

### 2.3. Test Animals

In line with commercial aquaculture practices that predominantly utilize all-male populations, this study used 150 one-year-old male GIFT tilapia (GIFT *Oreochromis niloticus*), with an average weight of 500 ± 50 g, obtained from the Beijing Xiao Tangshan Breeding Base. Prior to the experiment, muscle samples from five GIFT tilapia were tested to confirm the absence of SMZ residues. The GIFT tilapia were acclimated for one week in a recirculating aquaculture system, housed in a culture tank with dimensions of 1.5 × 1.5 × 1.0 m. During the experiment period, the water temperature was maintained at 22 ± 2 °C, continuous aeration was provided for 24 h, and natural light was utilized. One-third of the tank water was replaced every two days, and feed was administered accordingly. Extruded tilapia compound feed, produced by Zhanjiang Fumin Feed Co., Ltd. (Zhanjiang, China), was administered to the experimental group twice daily at 08:00 and 18:00. A ration equivalent to 1.5% of the total fish biomass was provided every time, and one hour after feeding, uneaten feed and excreta were promptly removed from the system. No feed was provided 24 h prior to the commencement of the experiment.

### 2.4. Dosing and Sampling

The dosage was determined by referring to the dosage in SC/T 1132-2016 “Code of Practice for the Use of Veterinary Drugs in Fisheries” [18] and considering actual production conditions. Multiple oral gavage tests on GIFT tilapia were carried out using SMZ at a dose of 100 mg/kg BW.

To prepare the oral gavage solution, SMZ was accurately weighed to 15.15 g and mixed with 25 g of feed powder in a 200 mL beaker. The mixture was thoroughly blended, followed by the addition of 150 mL of distilled water. The solution was stirred evenly to ensure homogeneity, and the final volume was adjusted to 200 mL with distilled water. The resulting mixed feed solution had a final SMZ concentration of 75 mg/mL.

Drug administration was performed using a 1 mL syringe for oral gavage. SMZ was administered to GIFT tilapia at a dose of 100 mg/kg BW. The initial dose was doubled to 200 mg/kg BW for the first gavage, and subsequent administrations were carried out once daily for 6 more consecutive days. Feeding was withheld during the entire 7-day administration period. Twenty-four hours after treatment cessation, standard feeding practices were reinstated. Following each gavage, the fish were monitored for regurgitation. Fish that exhibited regurgitation within 1 h were discarded from the experiment. After completing the oral gavage regimen, fish were sampled at the following time points: 0.33 days (8 h), 0.42 days (10 h), 0.5 days (12 h), 0.67 days (16 h), 0.83 days (20 h), and 1, 2, 3, 4, 6, 8, 10, 12, 16, 20, 25, and 30 days. At each time point, five fish were collected for analysis.

Blood samples were collected from the caudal vein using a 1 mL syringe. A total of 5 mL of blood was drawn and transferred into a centrifuge tube containing 1250 IU of sodium heparin. The samples were then centrifuged at 1790× *g* for 5 min at 4 °C. After centrifugation, the plasma was separated and stored at −20 °C for later analysis. Following blood collection, fish were euthanized by immersion in a solution of 300 mg/L tricaine mesylate (MS-222), and various tissues, including muscle, liver, kidney, skin, gills, and the remaining tissues (i.e., tissues other than those previously mentioned, mainly the visceral organs other than the liver and the intra-abdominal adipose tissue), were excised. Each tissue sample was placed in a separate, labeled, sealed bag and stored at −20 °C for subsequent analysis.

### 2.5. Sample Testing

The sample preparation and instrumental analysis methods were based on Announcement No. 1077-1-2008 of the Ministry of Agriculture of China [24]. The sample preparation procedure was conducted as follows: a 1 mL aliquot of plasma or 1 g of tissue (muscle, liver, kidney, skin, gills, or remaining tissues; the tissue samples were ground and homogenized) was transferred to a 10 mL centrifuge tube. To each sample, 50 μL of a 1 μg/mL internal standard SDM-D3 working solution was added and thoroughly mixed. Next, 5 mL of acidified acetonitrile was introduced, and the mixture was vortexed for 1 min followed by ultrasonic extraction for 10 min. After extraction, 1.0 g of Na_2_SO_4_ was added, and the mixture was vortexed for 1 min and then centrifuged at 1790× *g* for 5 min. The supernatant was carefully transferred to a new 10 mL centrifuge tube. A second extraction was performed by adding 4 mL of acidified acetonitrile, vortexing for 1 min, and centrifuging again. The supernatants from both extractions were combined and evaporated to dryness under a stream of nitrogen at 40 °C. The residue was reconstituted in 1 mL of a 20% methanol solution, followed by the addition of 2 mL of n-hexane for fat removal. The lower aqueous layer was collected, filtered through a 0.22 μm membrane, and subjected to LC-MS/MS analysis.

The chromatographic and mass spectrometric conditions were as follows: chromatographic column: C18 column (Hypersil Gold 150 × 2.1 mm, 5 μm) (Thermo Fisher, Waltham, MA, USA) and mobile phase: A, 0.1% formic acid solution (containing 5.0 mmol/L ammonium acetate); B, methanol; and C, acetonitrile. The mass spectrometry scanning mode was selected reaction monitoring (SRM), and the reaction monitoring ion pairs are shown in Table 1.

### 2.6. Method Validation

#### 2.6.1. Standard Curves

Appropriate volumes of the SMZ standard working solutions (0.05, 0.1, 0.2, 0.5, 1.0, 2.0, 5.0 10.0, and 20.0 μg/mL) were accurately pipetted. Fifty microliters of the SDM-D3 standard working solutions (1 μg/mL) were then added to those SMZ standard working solutions, and the volumes were adjusted to 1 mL with a 20% methanol solution. The mixtures were vortexed thoroughly to ensure uniformity. Subsequently, serial dilutions were performed to prepare standard solutions with concentration gradients of 0.05, 0.1, 0.2, 0.5, 1.0, 2.0, 5.0, 10.0, and 20.0 μg/mL. A standard curve was then constructed using the internal standard method.

#### 2.6.2. Limit of Detection (LOD) and Limit of Quantitation (LOQ)

A certain amount of the standard working solution was added to blank samples to prepare spiked tissues. For each concentration, five parallel experiments were carried out. Subsequently, the samples were processed according to the above-mentioned sample preparation method, and then detected by LC-MS/MS. The limit of detection (LOD) is defined as the spiked concentration at which the peak response of the spiked recovery sample reaches three times the noise level, while the limit of quantitation (LOQ) is determined as the spiked concentration where the peak response of the spiked recovery sample attains ten times the noise level.

#### 2.6.3. Determination of Recovery

A certain amount of the standard working solution was added to the blank samples, respectively, to prepare drug-containing samples with drug concentrations of 0.05, 0.5, and 5.0 μg/mL. Three replicates were set for each concentration. After processing the samples according to the above-mentioned preparation method, the samples were detected by HPLC-MS/MS, and the recovery rates of the drug in each tissue were calculated.

#### 2.6.4. Determination of Precision

For the spiked samples with concentrations of 0.05, 0.5, and 5.0 μg/mL, each concentration was measured five times within a single day to calculate the intra-day coefficient of variation. The inter-day coefficient of variation was determined by performing repeated measurements over a period of 5 days.

### 2.7. Data Processing

Data processing was carried out using Office 2019 Excel. The elimination curves were fitted using Origin 2019. The withdrawal period was calculated using the software WT1.4 [25], which calculated the time point at which the upper one-sided 95% tolerance limit for residue concentrations falls below the MRL of 100 µg/kg.

The following elimination formula was used to calculate the elimination parameters, following the methodology described by Chang et al. [26]:

(1) $$C_{t}=C_{0}\times e^{-\beta t}$$

C_t_ represents the concentration of SMZ in each tissue at time t, expressed in μg/kg or μg/L; C_0_ denotes the initial concentration, also expressed in μg/kg or μg/L; β is the elimination rate constant; and t represents time, measured in days (d).

The health risk of SMZ residues in tilapia was assessed using the estimated daily intake (EDI) and hazard quotient (HQ). EDI was calculated using the following formula, as applied in earlier study by Magna et al. [27]:

(2) $$\mathrm{EDI}=\frac{\mathrm{CD}\times \mathrm{DCAP}}{\mathrm{BW}}$$

where estimated daily intake (EDI) represented the calculated daily intake (μg/kg/d), consumption dose (CD) denoted the SMZ concentration in this study (μg/kg), daily consumption of aquatic products (DCAP) (kg/d) was derived from the Fifth Chinese National Nutrition and Health Survey (0.056 kg/d) [28], and body weight (BW) represented the average body weight of an adult (60 kg).

The hazard quotient (HQ) was used to evaluate the safety risk of the dietary intake of a harmful substance. The calculation formula is shown in the following equation:

(3) $$\mathrm{HQ}=\frac{\mathrm{EDI}}{\mathrm{ADI}}$$

where estimated daily intake (EDI) was derived from Formula (2), and acceptable daily intake (ADI) of SMZ (μg/kg/d) was set at 130 μg/kg/d [29].

## 3. Results

### 3.1. Method Validation Result

Within the range of 0.05–20 μg/mL for SMZ, the linear equation was y = 0.002x + 0.1794, with r^2^ = 0.9991, indicating an excellent linear relationship. The LOD of SMZ in tissues and plasma was 1 μg/kg or 1 μg/L, and LOQ was 2 μg/kg or 2 μg/L. At the three concentration levels in each tissue (0.05, 0.5, and 5.0 μg/mL), the average recovery rate of SMZ ranged from 77.9% to 108.5%. The intra-day coefficient of variation was less than 12%, and the inter-day coefficient of variation was less than 15%.

### 3.2. Elimination Patterns of SMZ in Various Tissues of GIFT Tilapia After Oral Gavage Admin-Istrations

**Figure 1:**
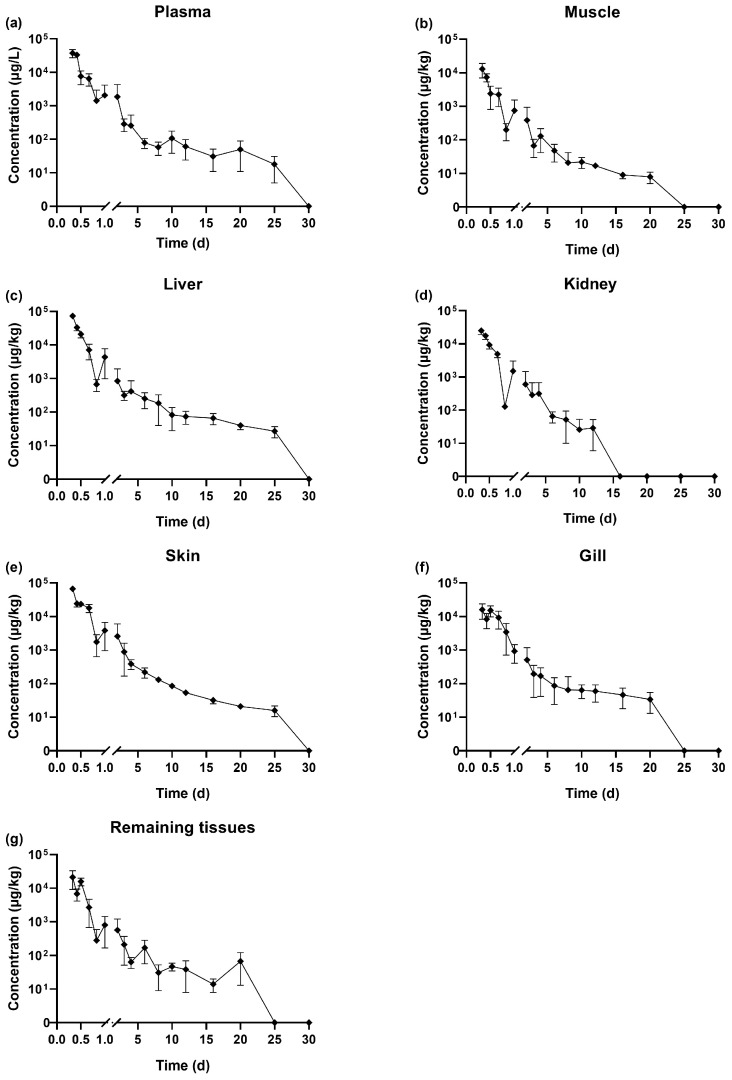
Elimination curve of SMZ in plasma (**a**), muscle (**b**), liver (**c**), kidney (**d**), skin (**e**), gill (**f**), and remaining tissues (**g**) in GIFT tilapia (*n* = 5).

The temporal changes in SMZ concentrations across various tissues following oral gavage administration are presented in Figure 1. It should be noted that concentrations reported as ‘undetectable’ were below the method’s limit of detection (LOD) of 1 μg/kg, not necessarily indicating a complete absence of the compound. The highest concentration of SMZ in all tissues was observed at the first sampling point, 0.33 days (8 h) post-administration. The concentration at this time point had the following order: liver (72,937 μg/kg) > skin (66,880 μg/kg) > plasma (37,377 μg/L) > kidney (25,127 μg/kg) > remaining tissues (21,167 μg/kg) > gill (16,106 μg/kg) > muscle (13,063 μg/kg).

At 0.33 days (8 h) after the cessation of drug administration, the SMZ concentration in the liver was approximately 5.58 times higher than in the muscle. The concentrations in plasma, kidney, and remaining tissues were similar, while the levels in muscle and gill were comparatively lower. From 0.33 to 0.83 days (8–20 h) post-withdrawal, SMZ concentrations in liver, skin, plasma, kidney, and muscle decreased, followed by a rebound between 0.83 days (20 h) and 1 day, then continued decline. In gill and remaining tissues, levels decreased from 0.33 to 0.42 days (8–10 h), rebounded between 0.42 (10 h) and 0.5 days (12 h), and declined thereafter. At 16 days post-withdrawal, SMZ in the kidney was undetectable first, followed by muscle, gill, and remaining tissues, which became undetectable by 25 days. In contrast, SMZ remained detectable in plasma, liver, and skin until 30 days after drug withdrawal.

The SMZ residue data at 0.33 days (8 h) post-drug withdrawal was fitted using Origin 2019, and the corresponding elimination curve equations were presented in Table 2. From these equations, it is evident that the elimination of SMZ follows a first-order kinetic model. As shown in Table 2, the β for SMZ in the plasma, muscle, liver, kidney, skin, gill, and remaining tissues of tilapia were 3.0093, 7.0295, 7.1029, 4.8745, 4.1390, 1.6153, and 4.8671, respectively.

The elimination half-lives were as follows: gill (0.4290 days, 10.30 h) > plasma (0.2303 days, 5.53 h) > skin (0.1674 days, 4.02 h) > remaining tissues (0.1424 days, 3.42 h) > kidney (0.1422 days, 3.41 h) > muscle (0.0986 days, 2.37 h) > liver (0.0976 days, 2.34 h). The half-lives of SMZ in the liver, kidney, and muscle were shorter compared to those in the gill and plasma. The liver exhibited the fastest elimination rate, with the highest elimination constant and the shortest half-life (0.0976 days). In contrast, the gill showed the slowest elimination rates, with a *t*_1/2_ of 0.4290 days, respectively. Notably, the longest half-life (in the gill, 0.4290 days) was 0.3314 days longer than the shortest half-life (in the liver, 0.0976 days), with the former being approximately 4.39 times longer than the latter.

### 3.3. Determination of the Withdrawal Period of SMZ in GIFT Tilapia After Multiple Oral Gavage Administrations

**Figure 2:**
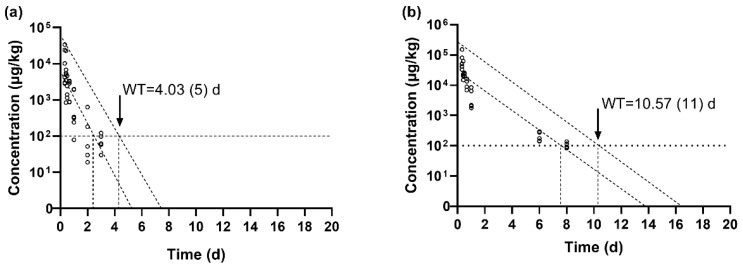
(**a**) Withdrawal period of SMZ in the muscle of GIFT tilapia; (**b**) Withdrawal period of SMZ in the skin of GIFT tilapia. The numbers in the brackets were obtained by rounding up the calculated values.

The maximum residue level (MRL) for total sulfonamides, including SMZ, in aquatic products is established at 100 μg/kg under Chinese regulation and EU legislation [14,15]. The findings of this study indicate that the SMZ concentration in muscle tissue declined below the MRL within 3 days, while in skin tissue, it took 10 days to fall below the MRL. The withdrawal periods for SMZ in tilapia muscle and skin were further calculated using WT1.4 software, with results presented in Figure 2. The estimated withdrawal period for SMZ in muscle was 4.03 days (Figure 2a), and in skin, it was 10.57 days (Figure 2b).

The time required for the SMZ concentration in muscle to fall below the MRL was 1.03 days shorter than the withdrawal period predicted by the software, representing a 25.56% discrepancy. For skin tissue, the difference was 0.57 days, with the experimental observation being 5.39% shorter than the software-derived withdrawal period. Under the experimental conditions of this study, and based on the 95% confidence interval and safety threshold, the software-predicted withdrawal periods were conservatively rounded up to the nearest whole number of days. Therefore, a withdrawal period of 11 days is recommended for SMZ in edible tissues (muscle and skin) of GIFT tilapia.

### 3.4. Dietary Exposure Risk Assessment of Sulfamethoxazole in Plasma and Tissues of GIFT Tilapia

**Figure 3:**
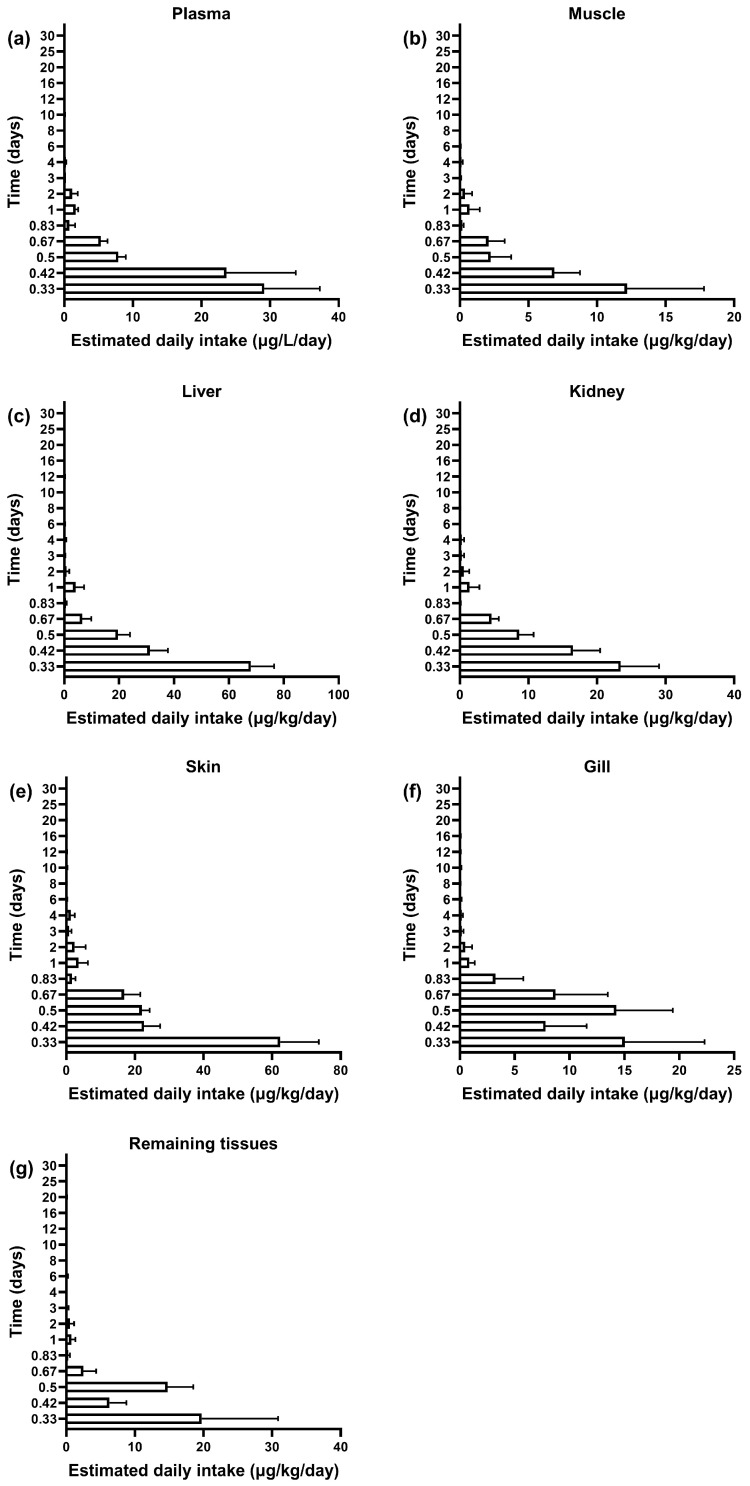
The profile of estimated daily intake of SMZ in plasma (**a**), muscle (**b**), liver (**c**), kidney (**d**), skin (**e**), gill (**f**), and remaining tissues (**g**) in GIFT tilapia (*n* = 5).

**Figure 4:**
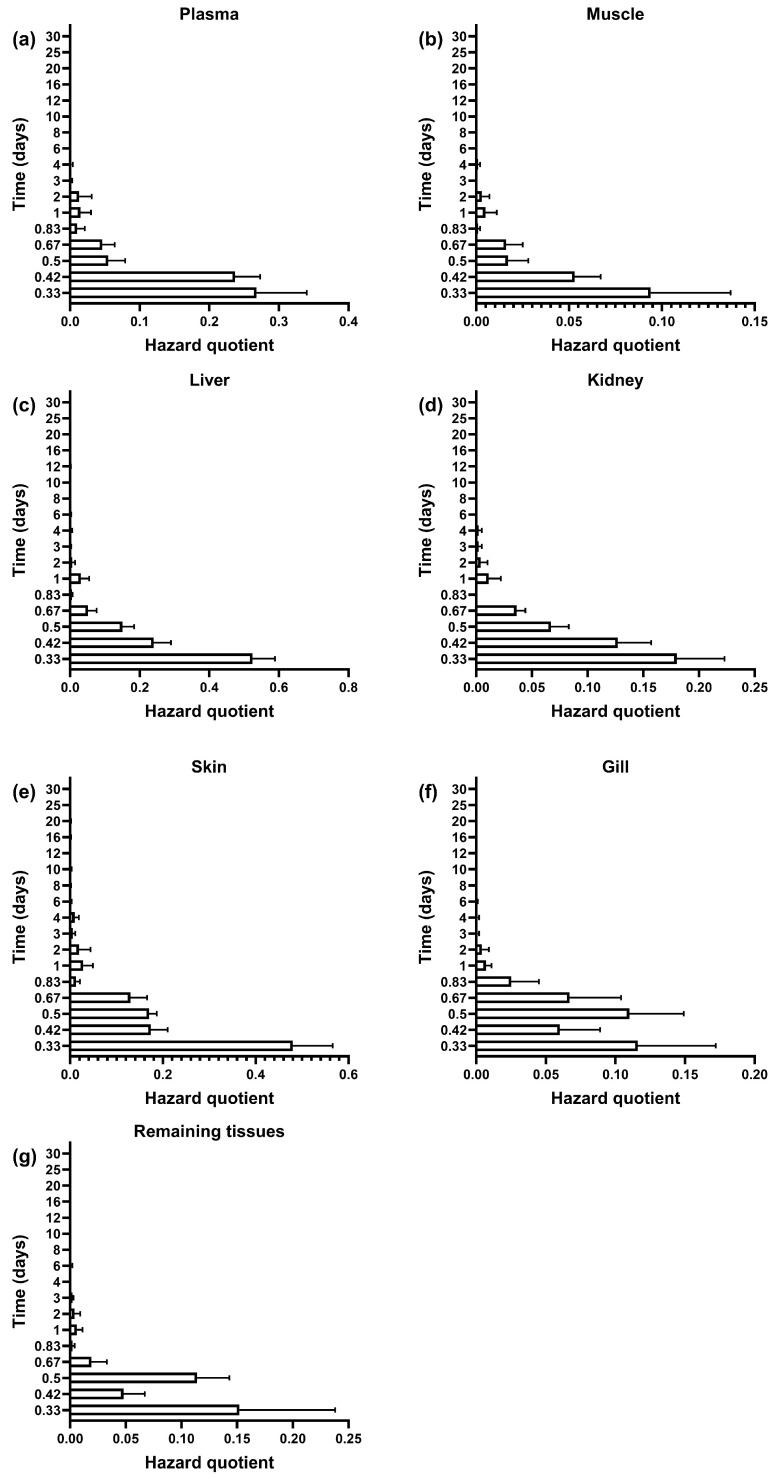
Hazard quotient of SMZ in plasma (**a**), muscle (**b**), liver (**c**), kidney (**d**), skin (**e**), gill (**f**), and remaining tissues (**g**) in GIFT tilapia (*n* = 5).

This study evaluated the residue dynamics and dietary exposure risk of SMZ in the plasma and tissues of GIFT tilapia using the estimated daily intake (EDI) and hazard quotient (HQ) (Figure 3 and Figure 4). Similar elimination trends were observed across all tissues: All tissues showed peak residue concentrations and maximum EDI at 8 h post-administration. Initial EDI values had the following order: liver (68.08 ± 8.40 μg/kg/d), skin (62.42 ± 11.20 μg/kg/d), plasma (34.89 ± 9.33 μg/L/d), kidney (23.45 ± 5.60 μg/kg/d), remaining tissues (19.76 ± 11.12 μg/kg/d), gills (15.03 ± 7.28 μg/kg/d), and muscle (12.19 ± 5.60 μg/kg/d) (Figure 3). After reaching the peak, the residue levels in all tissues rapidly declined. The EDI values dropped below 10% of the acceptable daily intake (ADI) at the following time points: 8 h for muscle, 12 h for plasma and kidney, 16 h for liver and remaining tissues, and 20 h for skin and gills.

The HQ values for all tissues remained below the safety threshold (HQ = 1) throughout the monitoring period. The values in descending order were as follows: liver (0.52 ± 0.07) > skin (0.48 ± 0.09) > plasma (0.27 ± 0.07) > kidney (0.18 ± 0.04) > remaining tissues (0.15 ± 0.09) > gills (0.12 ± 0.06) > muscle (0.09 ± 0.04) (Figure 4). Notably, although the liver exhibited the highest drug accumulation (68.08 μg/kg/d), its HQ value reached only 52.4% of the safety threshold. These findings suggest that, after continuous administration at a dose of 100 mg/kg BW for 7 days, the dietary exposure risk of SMZ in GIFT tilapia remained within a safe and controllable range.

## 4. Discussion

### 4.1. The Residue Elimination Patterns of SMZ in GIFT Tilapia

After 7 days of continuous SMZ administration, the highest concentration of SMZ in each tissue was detected at the first sampling point (0.33 d, 8 h). The concentrations of SMZ in various tissues, from highest to lowest, were as follows: liver, skin, plasma, kidney, remaining tissues, gill, and muscle. The liver’s highest SMZ concentration aligns with its role as the primary metabolic organ, consistent with sulfonamide pharmacokinetics in teleost [30]. The relatively low concentration of SMZ in muscle tissue may be attributed to the acetylation of sulfonamide drugs, which reduces their concentration in muscle. Zhang et al. also reported this phenomenon in their study on turbot (*Scophthalmus maximus*) [31]. Following drug withdrawal, the SMZ concentrations in various tissues exhibited a “decrease–increase–decrease” pattern. This cyclical trend may be due to reabsorption from the gut, specifically through enterohepatic circulation, which can lead to secondary absorption and, consequently, an increase in concentration [32]. The similar reabsorption phenomenon was also observed in the pharmacokinetic study of sulfadiazine in European eel (*Anguilla anguilla*), where the drug concentration in plasma and muscle increased during the reabsorption phase [33].

Tissue-specific detection limits were reached earliest in muscle (3 days) and latest in skin (30 days), reflecting differential clearance capacities. The SMZ concentrations in muscle and kidney were the first to fall below the limit of detection, with 3 and 6 days required, respectively. The remaining tissues and gill followed, reaching below the detection limit at 8 and 12 days, respectively. In most tissues, SMZ was eliminated below the limit of detection by day 25. However, in plasma, liver, and skin, SMZ remained detectable until day 30. The prolonged elimination of SMZ in skin tissue may be due to its large-scale accumulation from continuous oral gavage, as well as the relatively low blood flow in the skin and the slow elimination rate, making it more challenging to eliminate SMZ [32]. Similar findings have been reported by Stoffregen et al. [34], who observed a prolonged residue time of enrofloxacin (ENR) in the skin of Atlantic salmon (*Salmo salar*). Phu et al. [35] also reported that both SMZ and trimethoprim (TMP) had a longer residue time in the skin of catfish (*Silurus asotus*), consistent with the findings of this study.

In addition to skin tissue, the liver also exhibited relatively slow elimination of SMZ in this study, with detectable levels remaining up to 25 days. This may be due to the liver’s primary role in the metabolism of SMZ. Tourak [36] noted that sulfonamide drugs undergo acetylation in the liver of animals, a reversible process. The continuous acetylation and deacetylation of SMZ in the liver resulted in its accumulation, which hindered complete elimination. Wang et al. [37] observed similar patterns of residue and elimination for sulfadoxine in half-smooth tongue-sole (*Cynoglossus semilaevis*), with relatively low drug concentrations in the kidney and liver one day after the cessation of 100 mg/kg oral gavage for 3 days at a water temperature of 20 °C. This suggests that sulfonamide drugs may undergo acetylation in the liver and kidney of aquatic animals. Yuan and Ai [38] studied the residue and elimination patterns of SMZ in Nile tilapia (*Oreochromis niloticus*) after a single oral gavage of 100 mg/kg SMZ at a water temperature of 19 °C. Their findings showed that the residue concentration–time profile had the following order: plasma < muscle < liver, indicating a longer elimination time in the liver. Although the dosing regimen in their study differed from that in the present study, a similar trend was observed.

### 4.2. The Withdrawal Period of SMZ in GIFT Tilapia

Based on the MRL set by EFSA and China, the withdrawal period for SMZ was calculated using the WT1.4 software under the experimental conditions. The withdrawal periods in muscle and skin were 4.03 days and 10.57 days, respectively. Given the slower elimination rate of SMZ in fish skin, the withdrawal period should primarily consider the elimination time in the skin. Therefore, the withdrawal period for SMZ should be no less than 10.57 days. This is approximately half of the 500 degree-days recommended in the Fishery Drug Use Information Sheet [16]. Clearly, the currently specified withdrawal period for SMZ in China may be overly conservative.

The withdrawal period for fishery drugs is influenced by multiple factors, including the frequency and dosage of administration. Ju et al. [39] administered sulfamonomethoxine to GIFT tilapia at a dosage of 200 mg/kg under a water temperature of 28 ± 2 °C and calculated that the withdrawal period should be no less than 5 days. Compared to this study, although both studies involved sulfonamides, differences in dosage and administration frequency likely contributed to the variation in the calculated withdrawal periods.

In addition, various research studies have indicated that the method of drug administration can also significantly impact the withdrawal period of SMZ. Hou et al. [40] compared the oral gavage and medicated-feed administration of SMZ to *Carassius auratus* var. ‘Pengze’ at a dosage of 150 mg/kg for 10 consecutive days. Their results showed that the residual SMZ content in the tissues of the medicated-feed group was significantly lower than that of the oral gavage group. Additionally, the withdrawal period for the medicated-feed administration group was slightly shorter than that of the oral gavage group. This suggested that the administration method influences the length of the SMZ withdrawal period. Additionally, individual variations within the same species exist, where factors such as sex and age can influence drug residue elimination. This study did not account for the impacts of fish size (body weight) or sex differences on SMZ residue elimination dynamics. Therefore, future studies should incorporate weight and sex variables to refine withdrawal protocols, enabling the development of more detailed withdrawal period protocols tailored to these biological variables.

Temperature has also been shown to affect the withdrawal period of sulfonamides. Ju et al. [41] investigated the elimination and residue patterns of sulfamonomethoxine in GIFT tilapia at two different water temperatures (23 ± 2 °C and 28 ± 2 °C). After administering sulfamonomethoxine at a dosage of 200 mg/kg for 5 consecutive days, the study concluded that the withdrawal period should be no less than 140 h at 23 ± 2 °C and 120 h at 28 ± 2 °C. This indicates that higher water temperatures tend to shorten the withdrawal period. In this study, we selected a water temperature of 22 °C, which was relatively low for tilapia aquaculture. Under these conditions, the withdrawal period for sulfonamides was relatively long, providing a more conservative estimate that can better ensure food safety by reducing the risk of excessive drug residues.

Qu et al. [42] orally administered SMZ to turbot (*Scophthalmus maximus*) at a dose of 80 mg/kg body weight, finding that the elimination rate of SMZ in turbot was slow, and they recommended a withdrawal period of 27 days. Similarly, Han et al. [43] studied the residue elimination of compound SMZ in Songpu mirror carp, administering it at 50 mg/kg of body weight. They recommended a withdrawal period of no less than 15 days. These studies, which observed longer withdrawal periods under lower dosages and frequencies of administration, suggest that species-specific factors and metabolic differences play a role in determining the appropriate withdrawal period.

The aquaculture industry has diversity across the world, with vast species of aquaculture fish and a wide range of farming methods. However, a standardized approach to dosage and withdrawal periods is currently applied across the board for aquaculture drugs [3]. Given the significant physiological and metabolic differences between species [44,45], and the varied action mechanisms and metabolic pathways of different drug classes [46,47], this one-size-fits-all approach may not be suitable. Furthermore, factors such as the administration dosage, route of administration, and environmental conditions can significantly influence the distribution and elimination of drugs in fish, thereby affecting the withdrawal period [48,49]. Therefore, an accurate assessment of drug elimination patterns in tilapia requires consideration of factors such as the actual administration dosage, frequency, and environmental temperature, which better reflect real-world conditions. Such an approach would support food safety, facilitate international trade, and promote the sustainable development of aquaculture industry.

### 4.3. Potential Dietary Risk Assessment of Sulfamethoxazole in the Plasma and Tissues of GIFT Tilapia

Several studies have indicated that the long-term consumption of animal-derived foods containing antibiotic residues may lead to the accumulation of antibiotics in the human body, thereby posing potential health risks [50,51]. This study assessed the dietary risks of SMZ administration by calculating the estimated daily intake (EDI) and hazard quotient (HQ). The results showed that the initial average HQ values for all tissues at day 0.33 were below one, indicating that the exposure risk to SMZ remained within a safe range.

Several studies assessed the dietary exposure risk of SMZ in aquatic products. Liu et al. [52] evaluated the dietary exposure risks of sulfonamide drug residues in freshwater fish from Xianyang City and found that the International Food Safety Index (IFS) for sulfonamide drugs in market-sold freshwater fish was below one, indicating that the exposure levels were generally safe. Jansomboon et al. [53] conducted a risk assessment on catfish (*Silurus asotus*) and catfish products imported into Thailand, finding that the HQ values for SMZ were below one, suggesting that consuming catfish products did not pose adverse health effects. The results of this study are consistent with these findings. Based on this study and others, coupled with the rapid metabolism of SMZ and its relatively weak accumulation in animal-derived foods [54], the potential for SMZ to accumulate in aquatic products and pose a health risk is considered low.

Furthermore, studies on the residue of other antibiotics in fish also indicated that the potential dietary health risks caused by antibiotic use in aquaculture were minimal. Wang et al. [55] assessed the risk of antibiotic residues in pond-raised fish in Lingui District, Guilin, Guangxi, and found that the human health risk quotient (HQ) values were all below one, indicating a low risk of antibiotic exposure from consuming these fish. Luo et al. [56] analyzed 57 samples from 33 freshwater fish farms in Fujian Province, finding that long-term consumption of 90% of the sampled freshwater fish did not pose potential health risks to adults or children. Shi et al. [57] conducted a risk assessment on antibiotics in the muscle tissues of major fish species from Chaohu Lake, revealing that both the health risk quotient (HQ) and hazard index (HI) were below 0.1, suggesting that the health risk associated with consuming fish from Chaohu Lake was low. Despite the generally low risk quotients, it is important to note that the use of antibiotics in aquaculture could still potentially disrupt gut microbiota [58], contribute to antimicrobial resistance [59], and exacerbate environmental risks [60]. Therefore, attention should still be paid to the potential risks associated with the use of antibiotics such as SMZ in aquaculture.

### 4.4. Limitations and Future Perspectives

Our findings contribute to the understanding of SMZ elimination in tilapia and underscore the value of generating species-specific pharmacokinetic data. By demonstrating clear species-specific differences in drug metabolism, our work provides a case for developing withdrawal period guidelines that reflect the biological diversity within aquaculture, moving beyond one-size-fits-all recommendations.

While this study provides crucial species-specific data, it is important to acknowledge the context of its experimental design and its limitations. For instance, our use of oral gavage, while ensuring precise dosing for kinetic modeling, may not fully replicate farm settings where medicated feed is the common administration route. Future studies investigating SMZ kinetics following medicated feed administration would therefore provide data more directly applicable to commercial aquaculture.

The experimental temperature was maintained at 22 ± 2 °C. Although this lower temperature provides a conservative safety margin for withdrawal period calculation, future studies incorporating multiple temperature gradients would offer a more complete picture of temperature’s impact on SMZ elimination. Similarly, our analysis focused on the parent SMZ compound. As SMZ metabolites may contribute to the total residue load and possess distinct toxicological properties, future research characterizing their kinetics is highly warranted.

Further limitations relate to our modeling and sampling strategy. While the first-order kinetic model provided an excellent fit for key edible tissues (r^2^ > 0.95), its goodness-of-fit was lower for biologically complex samples like gills and remaining tissues (r^2^ = 0.73–0.83). Moreover, our sampling protocol deliberately commenced at 8 h post-administration to focus on the elimination phase critical for withdrawal period estimation, meaning the absorption peak (Tmax) was not captured. This design was appropriate for our primary objective, but future work aiming for a complete pharmacokinetic profile should incorporate earlier, more intensive sampling.

Finally, while this study provides crucial data for withdrawal period calculations, future risk assessments could be refined. Such assessments should consider potential exposure to metabolites, explore impacts on vulnerable human populations, and evaluate broader ecological implications, including contributions to antimicrobial resistance.

## 5. Conclusions

This study characterized the depletion of sulfamethazine (SMZ) in GIFT tilapia following a 7-day oral administration (100 mg/kg BW) at (22 ± 2) °C. SMZ was rapidly absorbed, reaching peak concentrations shortly after administration, and subsequently eliminated from various tissues at differing rates. While elimination half-lives varied among tissues, residues in edible muscle and skin tissues decreased to below the MRL (100 μg/kg) within 3 and 10 days post-withdrawal, respectively. Based on the slowest depletion observed in edible tissues (skin), a withdrawal period of 11 days is recommended for GIFT tilapia under these specific conditions to ensure compliance with established MRLs and safeguard consumer health. The calculated hazard quotients further supported that SMZ residues after this withdrawal period pose a low risk under standard consumption patterns. This work contributes to promoting responsible antimicrobial use and enhancing food safety in aquaculture. Future research should prioritize refining withdrawal period recommendations using more realistic administration methods and exploring environmental influences, while simultaneously expanding risk assessments to encompass vulnerable populations and critical ecological and public health impacts for advancing sustainable aquaculture.

## Figures and Tables

**Table 1:** Ion pairs for reaction monitoring.

Target Compound	Parent Ion *m*/*z*	Product Ion *m*/*z*	Collision Energy eV
Sulfamethoxazole	254	108	22
		156 *	16
Sulfadoxine-D_3_ (internal standard)	314	156 *	17

* Refers to quantitative fragment ions.

**Table 2:** Elimination curve equation and elimination parameter of SMZ in tissues of GIFT tilapia.

Tissue	Equation	β	t_1/2_ (d)	r^2^
plasma	y = 86,930.08e^−3.0093t^	3.0093	0.2303	0.8478
muscle	y = 148,547.29e^−7.0295t^	7.0295	0.0986	0.9562
liver	y = 851,754.91e^−7.1029t^	7.1029	0.0976	0.8829
kidney	y = 86,226.53e^−4.8745t^	4.8745	0.1422	0.8562
skin	y = 190,180.51e^−4.1390t^	4.1390	0.1674	0.8903
gill	y = 27,486.32e^−1.6153t^	1.6153	0.4290	0.7337
remaining tissues	y = 93,507.37e^−4.8671t^	4.8671	0.1424	0.8329

β is the final elimination rate constant, t_1/2_ is the elimination half-life, and r^2^ is the correlation coefficient.

## Data Availability

Relevant information is included in the article.
